# Atrial Rate And Rhythm Abnormalities In A Patient With Hyperkalemia

**Published:** 2009-05-15

**Authors:** Jonathan Rosman, Prashan Thiagarajah, Paul Schweitzer, Maurice Rachko, Sam Hanon

**Affiliations:** 1 Dept. of Cardiology, Beth Israel Medical Center. 16th street and 1st avenue. New York, New York 10003.

**Keywords:** Hyperkalemia, Atrial flutter, Conduction velocity, Membrane potential, Action potential

## Abstract

A 67 year old man presented with a serum potassium of 7.7 mEq/L and slow atrial flutter with variable A-V block and peaked T waves. Initial treatment for hyperkalemia was followed by an increase in the atrial flutter rate to 300 beats per minute. After hemodialysis the rhythm converted to sinus.

## Case Report

**Figure 1:**
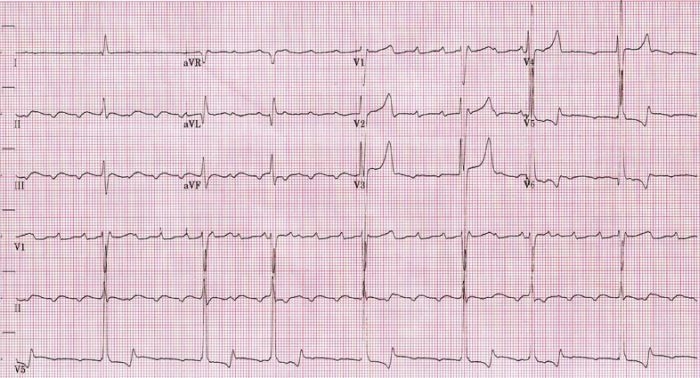
Admission ECG showing slow atrial flutter at an atrial cycle length of 400ms (atrial rate of 150bpm)

A 67 year old man with hypertension and chronic kidney disease on hemodialysis presented to the emergency department with a 2 day history of diarrhea and fatigue. He missed his last dialysis appointment because of his presenting symptoms. Physical exam revealed a blood pressure of 164/87 and irregularly irregular heart rate of 45 beats per minute (bpm). His ECG (Figure 1) showed slow atrial flutter (atrial rate ~160 bpm) with variable A-V block and peaked T waves. The patient had no known history of atrial arrhythmias and his ECG a week prior revealed normal sinus rhythm. Serum chemistry was significant for potassium of 7.7 mEq/L.  Echocardiography was significant for a left atrial diameter of  4.2 cm and a right atrial diameter of 4.7 cm.

**Figure 2:**
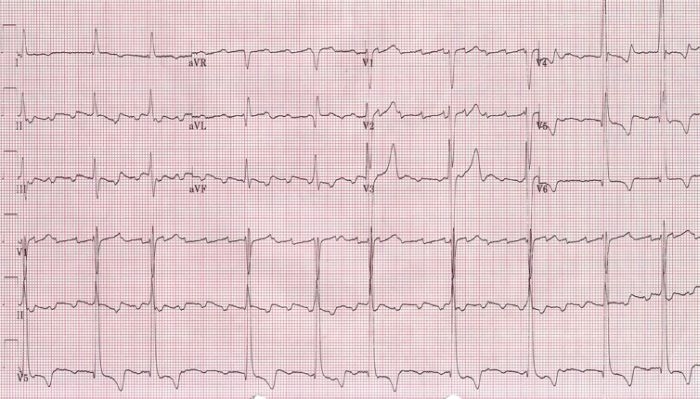
ECG following insulin and kayexelate showing a faster atrial flutter at an atrial cycle length of 280ms (atrial rate of 214 bpm)

**Figure 3:**
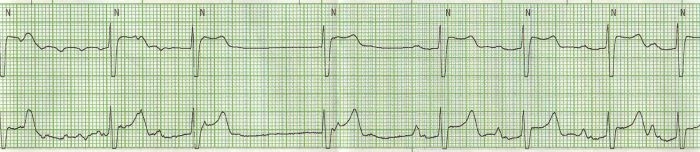
Telemetry strip 30 minutes after initiation of hemodialysis showing an atrial cycle length of 200ms (atrial rate of 300bpm) converting to normal sinus rhythm.

Initial treatment for his hyperkalemia included calcium gluconate, insulin and sodium polystyrene sulfonate (Kayexalate). Following this treatment, the atrial rate gradually increased to 214 bpm (Figure 2).  The patient was hemodialyzed for definitive treatment of hyperkalemia. Thirty minutes after initiation of hemodialysis, he converted to sinus rhythm (Figure 3).

## Discussion

Hyperkalemia produces a spectrum of ECG abnormalities and arrhythmias as a result of a range of effects on myocyte cellular electrophysiology [1]. Hyperkalemia reduces resting membrane potential (less negative), bringing it closer to threshold. Consequently, moderate hyperkalemia (6.0 -6.5 mEq/L) leads to a small increase in conduction velocity. As potassium levels continue to increase, the rate of rise of phase 0 of the action potential is reduced. This produces an overall slowing of intraatrial and intraventricular conduction [2-4].

The ECG series in our patient allowed observation of the rhythm abnormalities with different concentrations of serum potassium. The patient presented with an atrial flutter rate of 160 bpm when his serum potassium was 7.7 mEq/L, consistent with conduction slowing at potassium levels above 6.5mEq/L. As his serum potassium level decreased his atrial rate increased to 300 bpm, consistent with relatively faster conduction at lower serum potassium levels. In addition, the patient had no history of atrial arrhythmias and his atrial flutter resolved with normalization of his serum potassium. While ventricular arrhythmias and atrial fibrillation are occasionally seen with hyperkalemia, atrial flutter is rare [4,5].

## References

[R1] Fisch C (1973). Relation of electrolyte disturbances to cardiac arrhythmias. Circulation.

[R2] Nygren A (2000). Mathematical simulation of slowing of cardiac conduction velocity by elevated extracellular K^+^ in a human atrial strand. Ann Biomed Eng.

[R3] Whalley DW (1994). Voltage-independent effects of extracellular K+ on the Na+ current and phase 0 of the action potential in isolated cardiac myocytes. Circ Res.

[R4] Parham WA (2006). Hyperkalemia revisited. Tex Heart Inst J.

[R5] Genovesi S (2005). Prevalence of atrial fibrillation and associated factors in a population of long-term hemodialysis patients. Am J Kidney Dis.

